# Serum GP73, a Marker for Evaluating Progression in Patients with Chronic HBV Infections

**DOI:** 10.1371/journal.pone.0053862

**Published:** 2013-02-13

**Authors:** Hongshan Wei, Boan Li, Renwen Zhang, Xiaohua Hao, Yubo Huang, Yong Qiao, Jun Hou, Xin Li, Xingwang Li

**Affiliations:** 1 Beijing Ditan Hospital, Capital Medical University, Chaoyang District, Beijing, China; 2 Center of Lab test, 302 Military Hospital, Beijing, China; Yonsei University College of Medicine, Republic of Korea

## Abstract

This study was designed to investigate the role of serum GP73 for diagnosing significant fibrosis in patients with chronic hepatitis B virus (HBV) infections. Two populations were enrollment. All subjects were patients with chronic HBV infections. First population included 761 patients, who received liver stiffness measurement; the second population included 633 patients, who undertaken liver biopsy, in which 472 patients with nearly normal ALT. All patients received serum GP73 test. The effect of GP73 recombinant protein to HepG2 cells and LX2 cells were observed *in vitro*. Results showed that serum GP73 concentration is correlated with liver stiffness (r = 0.601). The area under ROC curve is 0.76. The sensitivity and specificity of GP73 for significant fibrosis (≥F2) diagnosis were 62.81%, 80.05% respectively (cut off: 76.6 ng/ml). Serum GP73 concentration was significantly correlated with the grading of fibrosis (r = 0.32, and 0.35, in 633 and 472 patients, respectively.) GP73 had a striking performance for diagnosing S2 in patients with chronic HBV infections. In 472 patients with nearly normal ALT, the sensitivity and specificity of GP73 for S2 diagnosis were 62.5% and 80.0% respectively, where the cut-off was set at 82 ng/ml. GP73 recombinant protein may prompt LX2 cells proliferation at the concentration 10–100 ng/ml. The present results indicated that GP73 may be a marker for diagnosing significant fibrosis in patients with chronic HBV infections, and may be a new contributor to fibrogensis.

## Introduction

Hepatic fibrosis, the common response associated with almost of all chronic hepatitis B virus (HBV) infection, ultimately leads to cirrhosis [1]. With great advancements in the antiviral therapy used for the treatment of chronic virus hepatitis, the accurate assessment of liver fibrosis is a vital need for successful individualized management. Current guidelines recommend antiviral therapy in chronic hepatitis B patients with significant fibrosis (≥2), whether or not ALT is abnormal [2]. Moreover, the significant fibrosis correlated strongly with poor clinical outcomes, compared with mild fibrosis [3]. Lack of accurate, reproducible and easily applied methods for fibrosis assessment is the major limitation in the clinical management. The current ‘gold standard’ for liver fibrosis detection is liver biopsy [4]. Liver biopsy can provide physicians useful clinical information, such as appropriate time to start antiviral therapy, predicting the response to treatment, assessing the natural course of hepatitis, and estimating prognosis of hepatitis. Although accuracy in detailed fibrosis classification may provide by liver biopsy; however, this method does has innate limits, such as invasive, sampling error and sample size effect, which limits its application. Consequently, the highly specific set of biomarkers for fibrosis grading always pursue by professionals.

In the past two decades, a number of markers in combination with clinical risk factors are used to evaluate fibrosis grade, such as FibroMeter, FibroTest, etc [5], [6], all being used for diagnosing significant fibrosis. In fact, the combination of several biomarker for evaluating fibrosis grading were better prognosis predictors than histological staging [7], [8]. Those methods have one common point, i.e., consisting of several serum marker and mathematical model used to calculate fibrosis index. Since all of those non-invasive models have moderate accuracy for determining significant fibrosis, new biomarkers for the diagnosis of significant fibrosis are still in strong demand by clinicians.

Recently, a novel Golgi protein, GP73, was used to diagnosing hepatocelullar carcinoma [9]. This protein initially reported by Kladney RD, et al [10], and they also found that GP73 expression is increased in cultured cells by viruses infection [10], Subsequently, several investigations demonstrated that GP73 protein is overexpressed in a variety of acute and chronic liver diseases [11], and serum concentration correlated with progression of chronic liver disease [12], [13]. However, the relationship between serum GP73 concentration and staging or grading of chronic liver disease is still a pendent question. The present study was designed to evaluate the serum GP73 for diagnosing significant fibrosis and liver cirrhosis.

## Materials and Methods

### Study design

This study registered at ChiCTR.org (No.DDT-11001397) Oct, 2010, and included two populations. First population consisted of 761 patients with chronic hepatitis B, who were received liver stiffness measurement; second populations involved 633 patients with chronic HBV infections, in which 472 patients with nearly normal ALT (<80 U/L). Patients in second populations were received liver biopsy and pathological examination. All patients consecutively admitted to two centers (Beijing Ditan Hospital and 302 Military Hospital), between Aug. 2010 and Mar.2012. The study was approved by the Institutional Review Board of the Beijing Ditan Hospital, Capital Medical University. For group enrollment, liver stiffness measurement or liver biopsy were based on clinical requirement. Before initiating drug therapy, the serum samples were collected, and stored at −70°C.

### Biochemical analysis

The liver function tests including serum albumin, total bilirubin (TB), and alanine aminotransferase (ALT) were measured using a Roche Hitachi 717 chemistry analyzer at the central laboratory of Beijing Ditan hospital. Quantitative determination of GP73 in serum was performed using commercially available enzyme-linked immunosorbent assay (ELISA) (Hotgen Biotech Inc., Beijing, China), according to the manufacturer's protocol.

### Transient elastography measurement

Liver stiffness was measured with a FibroScan® device (FibroScan®, Philips, France), based on manufacturer's protocol. Results were expressed in kilopascals (kPa). Ten successful acquisitions were performed for each patient, and the median value was calculated by the device. The cut-off point of liver stiffness score for significant fibrosis, and liver cirrhosis referenced to the previous report [14], [15] i.e. 8.8 kPa(F2), for significant fibrosis, 16.9 kPa for liver cirrhosis.

### Liver biopsy and Immunohistochemistry

Liver biopsies were obtained using 16G disposable needles (Hepafix, Germany). Fibrosis staging was considered reliable when the liver specimen length was ≥1.5 cm or the portal tract number ≥10. Liver specimens were stained with Masson trichrome and interpreted by two highly experienced liver pathologists. Liver fibrosis was scored on a 0–4 scale according to the METAVIR scoring system [16]. For GP73 staining, 3–5 µm formalin-fixed, paraffin-embedded samples were dewaxed and rehydrated. After slides incubating in 3% hydrogen peroxide, sections were incubated with GP73 antibody (HotGen Biotech, Beijing, China) overnight at 4°C; HRP-labeling anti-rabbit (Boster Bio., Wuhan, China) were used as secondary antibodies. 3,3′-Diaminobenzidine (DAB) Substrate Chromogen System (Dako) and was employed in the detection procedure. Images were acquired on an Olympus E520 (Tokyo, Japan) microscope.

### Cell culture and proliferation assay

Hepatoma cell line (HepG2) was reserved in our laboratory. Hepatic stellate cell line (LX2) was conferred by Prof. Cheng (Insititute of Infectious Disease, Capital Medical University). LX2 cells line is a widely used hepatic stellate cell in the fibrosis investigation [17]. HepG2 and LX2 cells were cultured at 37°C in a humidified atmosphere containing 5% CO_2_ in Eagle's minimum essential medium supplemented with10% fetal bovine serum. The ultimate concentration of GP73 recombinant protein added in supernatant was 1.0, 10.0, 20.0, 50.0, and 100.0 ng/ml respectively. After 48 hours coculturing, cell proliferation was evaluated with OD value, which was detected by CCK8 assay kit (Dojindo, Kumamoto, Japan), based on manufacture's protocol.

### Western blot

Western blot was performed with standard protocol. Briefly, after cells cocultured with GP73 recombinant protein 48 hours, whole-cell extracts were prepared in assay buffer containing a protease inhibitor cocktail. Protein assays were performed using a BCA Protein assay kit (Pierce/Thermo Scientific, USA) according to the manufacturer's instructions. Total protein was electrophoresed in SDS–PAGE gels, and transferred to nitrocellulose membranes and then blocked with 5% milk in PBS, pH 7.4 with 0.05% Tween-20, incubated with collagen I or collagen III polyclonal antibody (Santa Cruz, USA) and anti-rabbit secondary antibody conjugated to horseradish peroxidase (Santa Cruz., USA). GP73 was detected by chemiluminescence.

### Statistical analysis

Statistical analysis was performed using GraphPad Prism 5.0. Student *t* test was used to compare the difference of serum GP73 concentrations between different patients groups (mild and significant fibrosis group). Correlation between serum GP73 concentration and liver stiffness scores were calculated using Pearson's correlation coefficient (r). Data were expressed as mean ± SEM. P-values <0.05 were considered to be statistically significant. With liver stiffness value (FibroScan) or biopsy as the “gold standard”, the diagnostic performance of GP73 was evaluated by performing the Area under the ROC curve (AUROC) with 95% confidence interval (CI). For adjusting other confounders (Sex, Age, ALT, Total Bilirubin, Albumin, Platelet), we performed multivariate ordinal logistic regression analysis by SPSS 16.0.

## Results

### Patient's characteristics

From Aug. 2010 to Mar 2012, 761 patients received liver stiffness measurements; 633 patients received liver biopsy, in which 472 patients with nearly normal ALT. Those patients consecutively admitted into Beijing Ditan Hospital, Capital Medical University and 302 Military Hospital. The demological materials of two populations were showed in table 1.

**Table 1:** Patient's clinical characteristic.

Parameter	Description	
Group	FibroScan (761)	liver biopsy (633)
Sex/age		
Male	492 (39.48±12.32 yrs)	440(36.11±10.72 yrs)
Female	269 (40.62±13.76 yrs)	193(34.07±10.37 yrs)*
Clinical diagnosis		
Chronic hepatitis	649 (85.28%)	582(91.94%)
Liver cirrhsis	112 (14.72%)	51(8.06%)
Comorbidity		
Diabetes	48(6.31%)	17(2.69%)
Hypertension	11(1.45%)	7(1.11%)
Coronary heart disease	4(0.53%)	2(0.32%)
HBeAg		
Positive	436(57.29%)	397(62.72%)
Negative	325(42.71%)	236(37.28%)
HBV DNA		
< 2 log	68(8.94%)	36(5.69%)
≥ 2 log	693(91.06%)	597(94.31%)
BMI (Kg/m^2^)		
Male(mean ± S.D.)	22.60±5.12	24.26±6.67
Female(mean ± S.D.)	20.73±4.93*	21.33±3.91*
Total bilirubin (μmol/L)	22.79±38.0	21.30±37.54
Albumin (g/L)	43.36±6.11	42.86±6.06
Prothrombin time (s)	12.88±2.20	12.71±9.35

* Compared with male group, p<0.05.

Since without any patients with ascites, no related information was showed.

### Serum GP73 concentration significantly correlated with hepatic stiffness

**Figure 1:**
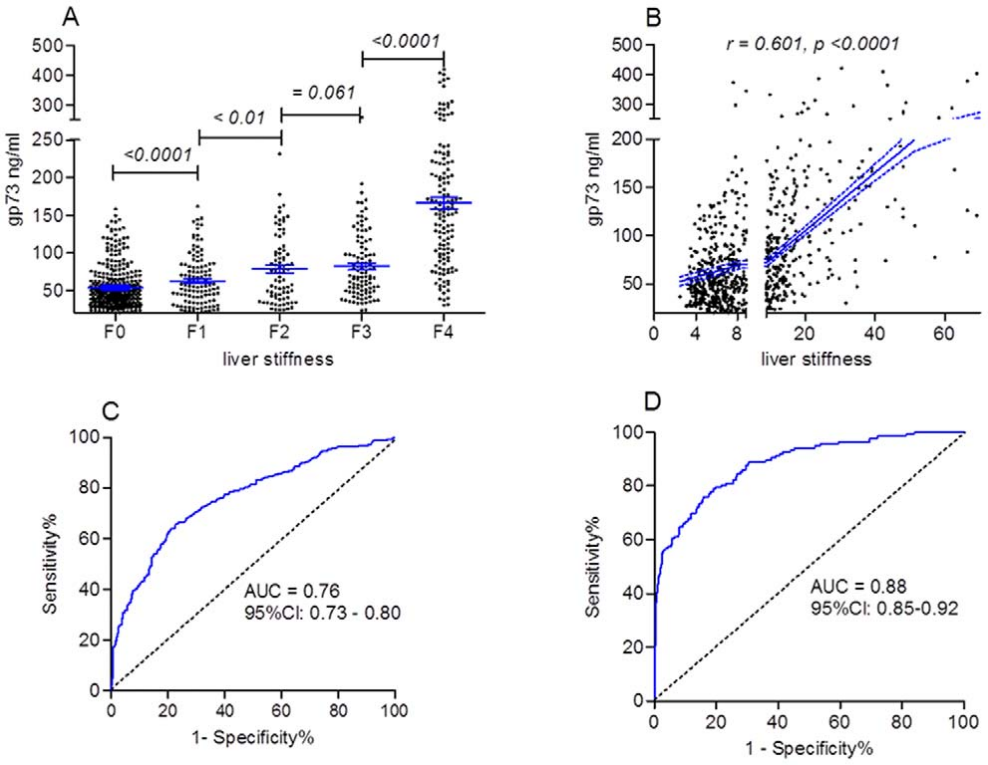
Serum GP73 concentration was correlated with liver stiffness (761 patients). A: Different GP73 levels were observed in patients with different groups of liver stiffness. B: serum GP73 concentration was correlated with liver stiffness. C and D: the ROC analysis of GP73 was performed on diagnosis of significant fibrosis and liver cirrhosis. The numbers after symbols “<”or “ = ” are p value.

Based on more recently report, the diagnostic thresholds of liver stiffness in discriminating fibrosis stages ≥ F1, ≥ F2, ≥ F3 and  =  F4 were 7.1 kPa, 8.8 kPa, 10.7 kPa, and 16.9 kPa, respectively. Based on data of liver stiffness, 57.95% (441/761) patients had mild fibrosis (324 patients with very mild fibrosis; 117 patients with F1 grade). 42.05% (320/761) patients were significant or severe fibrosis (F2:79, F3:105, F4:136). Obviously, more cirrhotic patients were confirmed with liver stiffness than initially clinician's diagnosis (Fig. 1A, Table 2).

**Table 2:** Serum GP73 concentration related with liver stiffness ( FibroScan), ALT, Fibrosis grading, and serum HBV DNA.

Parameter	N	GP73(ng/mL)	
		Mean ± SD	95%CI
Liver stiffness^a^			
< F1	324	53.70±28.84	50.55–56.85
F1	117	62.54±34.31	56.27–68.60
F2	79	78.46±45.35	68.30–88.61
F3	105	91.90±44.51	73.28–90.51
F4	136	166.0±87.39	151.2–180.8
Fibrosis grading^b^			
0.5	48	56.75±38.33	45.62–67.88
1.0	232	60.37±40.07	55.19–65.55
1.5	35	72.03±37.75	59.06–84.99
2.0	57	80.03±59.48	64.25–95.81
2.5	26	93.80±49.04	73.99–113.6
3.0	40	92.28±69.69	68.10–120.7
3.5	18	94.40±52.88	68.10–120.7
4.0	16	458.1±119.1	94.67–221.6
ALT(U/L)^c^			
≤ 40	274	71.90±55.28	65.33–78.48
< 80	197	72.52±53.07	65.08–79.98
80–200	93	88.35±77.53	72.38–104.3
> 200	69	137.0±89.25	115.5–158.4
HBV DNA(log10 IU/ml)^b^			
negative	25	49.13±17.39	41.95–56.31
< 4	99	65.00±41.52	56.72–73.29
≥ 4	106	91.88±88.7*	74.79–109.0
≥ 6	194	81.93±63.81	72.89–90.96
≥ 8	48	83.08±58.15	66.20–99.97

a **.** 761 patients received liver stiffness measurements; **^b^.** 472 patients with nearly normal ALT; ^c^. 633 patients with chronic hepatitis B infections; * *P*<0.05 Compared with patients with HBV DNA less than 4 Log.

To ascertain the correlation between serum GP73 concentration and liver stiffness, we firstly performed correlated analysis. Results showed that serum GP73 concentration is correlated with the value of liver stiffness (Correlation coefficient *r* = 0.601, p<0.0001). (Fig. 1B) Serum GP73 concentrations were significantly different between patients with different fibrotic group, except for F3 group (Fig. 1A). The important is that GP73 levels in patients of F2 group (78.46±45.35 ng/ml) is significantly higher than those of in patients of F1 group (62.54±34.31 ng/ml, p<0.01). This result suggested that GP73 concentration may be a marker for differentiating significant fibrosis (≥ F2) with mild fibrosis (<F2). The other interesting result is, for most cirrhotic patients, serum GP73 concentrations were especially higher than patients in F2 or F3 group. Despite serum GP73 concentration in patients of F3 group (81.90±44.51 ng/ml) were higher than those of patients in F2 group (78.46±45.35 ng/ml), this difference is no significant.

To clarify the optimal cut-off value of GP73 in diagnosing significant fibrosis (≥F2) in patients with chronic hepatitis B, we performed receiver operator characteristic (ROC) curve analysis. ROC curve analyses showed that the sensitivity and specificity of GP73 for significant fibrosis diagnosis were 62.81% (95% CI: 57.26%–68.12%), 80.05% (95% CI: 80.05%–83.68%) respectively, where the cut-off value was set at 76.6 ng/ml. The area under ROC curve is 0.76 (95% CI: 0.73–0.80). The positive predictive value (PPV), the negative predictive value (NPV), and acuuracy were 74.73%, 67.69, and 72.01%, respectively. If the diagnostic cut-off value was set at 135.4 ng/ml, the sensitivity and specificity of GP73 for diagnosing liver cirrhosis (F4) were 60.29% (95% CI: 51.55%–68.58%), 94.01% (95% CI: 91.84%–95.75%) respectively. The area under ROC curve is 0.88 (95%CI: 0.85–0.92). (Fig. 1.C, D). The PPV, the NPV, and acuuracy were 91.68%, 60.29%, and 86.07%, respectively.

### Sensitivity and specificity of GP73 for diagnosis significant fibrosis

**Figure 2:**
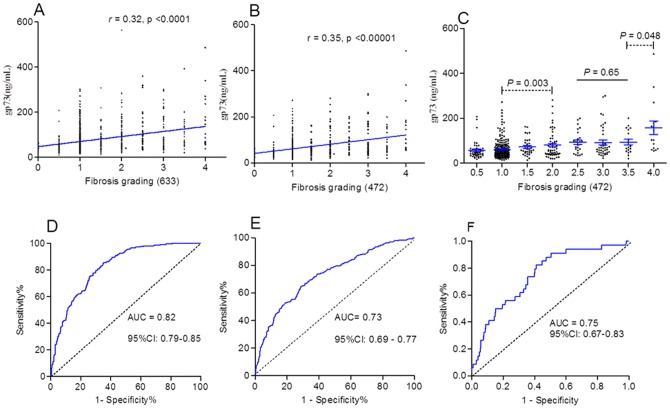
Serum GP73 was correlated with grading of patients. A: serum GP73 was correlated with grading of 633 patients. B and C: serum GP73 was correlated with grading of 472 patients with nearly normal ALT. D, E, F: ROC analysis of GP73 was performed on diagnosing S2(D), G2(E), and cirrhosis (F) respectively.

Serum GP73 concentration was significantly correlated with the grading of fibrosis (correlation coefficient *r* = 0.32, and 0.35, in 633 patients with chronic hepatitis B, and in which 472 patients with nearly normal ALT, respectively.) (Fig. 2.A, B). The mean GP73 concentration increased with liver grading aggravation, but significantly statistical differences only observed in several groups (Table 2; Fig. 2C).

To characterize GP73 as a new diagnostic tissue marker of significant fibrosis (≥S2), or moderate/severe inflammation (≥G2), we conducted a ROC analysis. The results showed that GP73 had a striking performance for diagnosing S2 or G2 in patients with chronic HBV infections. In patients with nearly normal ALT, the sensitivity and specificity of GP73 for S2 diagnosis were 62.5% (95%CI: 56.26–68.45%) and 80.0% (75.9–83.68%) respectively, where the cut-off was set at 82 ng/ml (Fig. 2D); The PPV, NPV, and diagnosing accuracy were 87.76%, 67.98%, and 80.29%, respectively. For G2 diagnosis, the sensitivity and specificity were 53.2% (95% CI: 47.35–58.95%) and 80.21(75.89–84.05%) respectively, the cut-off was 85 ng/ml (Fig. 2, E). The PPV, NPV, and diagnosing accuracy were 73.11%, 54.78%, and 68.64%, respectively. If the cut-off was set at 138.4 ng/ml for cirrhosis (S 3.5–4) diagnosis, the sensitivity and specificity were 38.24% (22.17–56.44%) and 90.18% (87.00–92.80%) respectively (Fig. 2.F). The PPV, NPV, and diagnosing accuracy were 94.95%, 23.21%, and 86.44%, respectively.

### Which factors related with serum GP73 levels?

**Figure 3:**
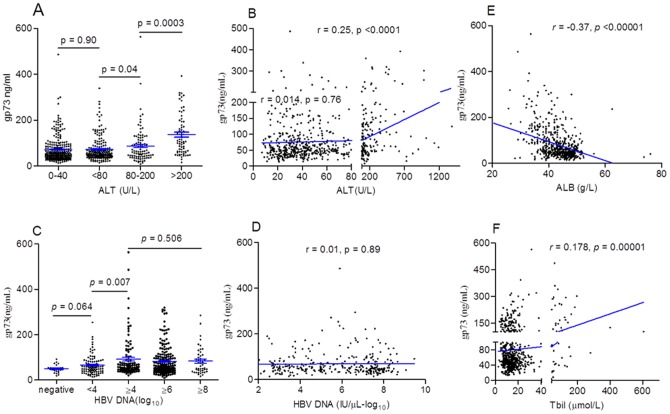
Serum GP73 concentration was related with levels of different biochemical marker. A and B: serum GP73 concentration was correlated with ALT in patients with ALT ≥ 80 U/L, but nearly normal ALT was not. Although different HBV DNA levels had their different GP73 concentration (C), the correlation was not significant (D). Sample number may be one of most important causes. GP73 were also correlated with total bilirubin (F), especially, significantly correlated with serum ALB negatively (E).

Multivariate ordinal logistic regression analysis was performed to adjust other potential confounders (Sex, Age, ALT, total bilirubin, albumin, Platelet). The results showed that serum GP73 was an independent risk factor of liver fibrogenesis in CHB patients with nearly normal ALT. The relative risk was increased 1.106 with the serum GP73 increasing 10 ng/mL. The results were showed in table 3. To explore the causes of GP73 increasing in serum, we further performed a Pearson's correlation analysis on several biomarkers, which reflected virus replication, hepatocytes injury, and other liver functions. As Fig. 3A and 3B showed, that ALT, total bilirubin (Tbil) were positively correlated with serum GP73 concentration. The correlation coefficient *r were 0.25, 0.18*, respectively (Fig. 3, B, F). The interesting data was that ALT seemed not significantly correlated with GP73 concentration in 472 patients with nearly normal ALT (r = 0.014, p = 0.76), by contraries, the total of 633 patients was (r = 0.25, p = 0.0003) (Fig. 3B, Table 2). Similarly, although HBV DNA was not correlated with serum GP73 concentration (r = 0.01, p = 0.89), the serum GP73 concentration of patients with HBV DNA above 4log was significantly higher than those of patients with HBV DNA below 4 log (p = 0.007) (Fig. 3C and 3D; Table 2). The other interesting result was that patient's GP73 levels were negatively correlated with their ALB levels (Fig. 3E).

**Table 3:** The Multivariate ordinal logistic regression analysis for the factors assocaited with Fibrogenesis.

Parameter	*b*	*stb*	*Wald χ^2^*	*P*	*OR*	*95%CI for OR*	
						*Lower*	*Upper*
Fibrosis grading							
4	0.25	0.88	0.08	0.771	-	-	-
3.5	1.22	0.86	2.02	0.155	-	-	-
3	2.21	0.86	6.62	0.010	-	-	-
2.5	2.64	0.86	9.39	0.002	-	-	-
2	3.37	0.87	15.17	<.0001	-	-	-
1.5	3.73	0.87	18.41	<.0001	-	-	-
1	6.59	0.91	52.65	<.0001	-	-	-
0.5	8.00	0.95	70.86	<.0001	-	-	-
GP73 (per 10 ng/mL)	0.01	0.00	30.62	<.0001	1.010	1.007	1.014
ALB (per 10 g/L)	−0.08	0.02	18.03	<.0001	0.927	0.895	0.960
PLT (per 10 ×109/L)	−0.01	0.00	30.87	<.0001	0.992	0.990	0.995

Note: Adjusted the factors including Sex, Age, ALT, total bilirubin, albumin, and platelet.

**Figure 4:**
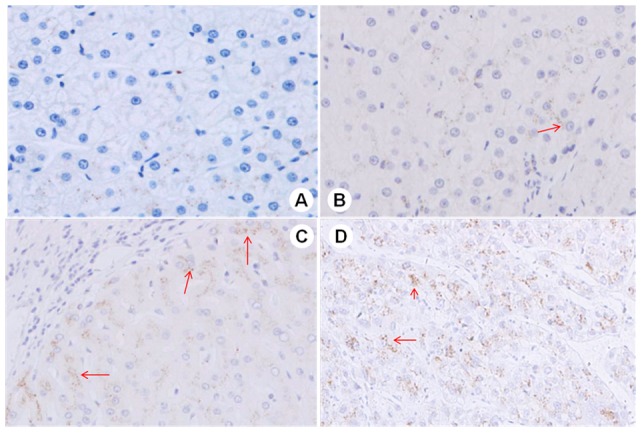
GP73 were stained in different liver tissue. GP73 was stained in brown. Arrow indicated positive cells. A: mild fibrosis (S1); B: significant fibrosis (S2); C: severe fibrosis (S3–4); D: cirrhosis (S4).

To further validate GP73 expression in liver tissue with different fibrotic gradings, we observed character of GP73 staining in different biopsy sample. Immunohistochemical analysis showed that GP73 positive cells mainly scattered in parenchymal cells, but several non parenchymal cells also positive staining (Fig. 4). This result was consistent with Iftikhar R, et al. [12], report. Compared with mild fibrotic tissue, GP73 was strongly expressed in significant or severe fibrotic liver tissue.

### Serum GP73 may be a contributor to liver fibrosis

**Figure 5:**
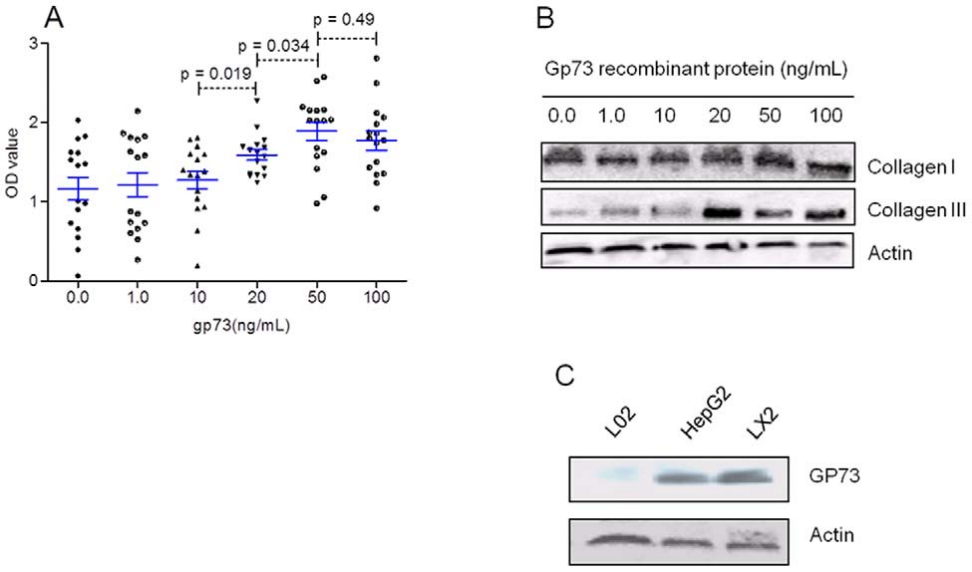
Gp73 recombinant protein prompted LX2 cells proliferation. A: when the concentration of GP73 recombinant protein was above 20 ng/ml, the LX2 proliferation was prompted. B: GP73 recombinant protein up-regulated collagen III expression, but collagen I was not. C: GP73 expression evaluated in different cells *in vitro*.

To investigate the effect of GP73 to hepatocytes or hepatic stellate cells, we used different concentration of GP73 recombinant protein (1.0, 10.0, 20.0, 50.0, and 100.0 ng/ml) coculturing with HepG2 cells, or LX2 cells. The result showed that GP73 may obviously prompt proliferation of LX2 cells (Table 4; Fig. 5A), but without any effect on HepG2 cells *in vitro* (data not show). With concentration of GP73 recombinant protein increasing (from 10 ng/mL to 80 ng/mL), the OD values of cultured LX2 cells also increased (Fig. 5A). The results suggested that GP73 recombinant protein may prompt LX2 cells proliferation *in vitro*. After cocultured 48 hours, the collagen III expression in LX2 cells was increased, but the collagen I was not (Fig. 5B). We speculated that GP73 might regulate hepatic stellated cells by autocrine, since LX2 also expressed GP73 *in vitro* (Fig. 5C).

**Table 4:** Effects of gp73 recombinant protein on LX2 cells.

GP73 recombinant Protein (ng/ml)	N	OD value	
		Mean ± SD	95%CI
0.0	16	1.17±0.58	0.86–1.48
1.0	16	1.22±0.61	0.90–1.54
10.0	16	1.27±0.44	1.04–1.51
20.0	16	1.59±0.27	1.45–1.73
50.0	16	1.89±0.46	1.64–2.13
100.0	16	1.77±0.48	1.52–2.03

## Discussion

The ultimate aim of fibrosis grading is provided clinicians with accurate information for treatment decision and prognosis judgment. Identifying significant fibrosis is also one of critical factors for treatment decision, especially for patients with mild abnormal ALT [18]. Avoided or reduced times of liver biopsy, but obtained pathological information from liver tissue, is always pursued by clinicians. Multi-marker combination can provide more accurate information about fibrosis [19], but result in increasing the patient's expenditure and clinician's working load. Based on our present data, GP73 might be a useful single marker for diagnosing significant fibrosis and cirrhosis in patients with chronic HBV infections.

The first question is why serum GP73 concentration correlated with liver stiffness? Based on recently reports, serum GP73 concentration related with progression of chronic liver diseases [13], [20]. Different with other HCC marker, increased serum GP73 is related to hepatic impairment and chronic fibrosis [21], [20]. In patients with Wilson disease, serum GP73 levels were associated with liver inflammation, fibrosis, and dysplasia, rather than copper overload [22]. More importantly, other experimental research showed that hepatic stellate cells are also expressed GP73 [23]. This result consistent with our data, and indicated that more hepatic stellate cells activation, more significant fibrosis, and resulting in serum GP73 more increasing.

Strict adherence to practice guidelines of chronic hepatitis B, will make a number of patients with nearly normal ALT lost opportunities of receiving antiviral therapy. In fact, recommended ALT thresholds may not absolutely reflect disease activity or degree of fibrosis [24]. More importantly, significant fibrosis (≥F2, or S2), or moderate hepatocytes injury (G2) are markers for beginning antiviral therapy in patients with chronic hepatitis B, based on present guideline [25]. Compared with other multi- parameter prediction models for grading fibrosis, GP73 is a single marker, which can be analysis with general enzyme-linked immunosorbent method. This new marker may be conveniently used in clinical practice, especially in developing countries for differentiating significant fibrosis with mild fibrosis in patients with chronic hepatitis B.

Liver stiffness is believed one of best non-invasive methods for evaluation liver fibrosis stage and disease progression. However, one question is what optimal cut-off value being chosen for fibrosis grading. Because numerous investigations provided different cut-off value for liver fibrosis classification, it was difficult to select optimal grading standard [26]. Based on recently reports, different research team presented different cut-off value for diagnosing significant fibrosis. Guha IN, et al [27], Stabinski L. et al [28], and Fung J, et al [29], presented 8.8 kPa, 9.3 kPa, 8.1 kPa respectively as optimal cut-off value for diagnosing significant fibrosis (≥F2). Since too higher cut-off value may be to lower the diagnostic sensitivity, we selected the relatively higher cut-off value, 8.8 kPa, for diagnosing significant fibrosis, in order to increase diagnostic specificity and accuracy. Difference of body constitution between east and west countries is other factor in our consideration, because liver stiffness variation in different populations [30]. Based on our present results, significant statistical differences only observed in several groups, although serum GP73 concentrations increasing with fibrosis progression. We speculated that these phenomena may be, at least in part, result in numbers of sample.

Based on data of stiffness measurement, setting 76.6 ng/ml as cut-off value may be appropriate for significant fibrosis diagnosis in chronic hepatitis B population. The impressive finding of this study was a obvious difference in GP73 concentration in patients with different fibrotic grading, especially in patients with nearly normal ALT (Table 2). According to results of liver biopsy, 80.21 ng/ml and 85 ng/ml, may effectively differentiate significant fibrosis (S2) or moderate injury (G2) from mild fibrosis or injury respectively. Integrating all abovementioned results, we proposed that 85 ng/ml may be an appropriate cut-off value for diagnosing significant fibrosis of moderate/severe hepatocytes injury from patients with chronic HBV infections. If the cut-off value was set at 135 ng/ml, GP73 was also a potent marker for diagnosing liver cirrhosis.

Although GP73 (tr/tr) mice (with a severe truncation of the GP73 C-terminus) developed marked abnormity in liver, the role of GP73 in liver disease is still unknown [31]. The other interesting result is that GP73 may be not only a fibrosis marker, but also a contributor to fibrogenesis in patients with chronic HBV infections. Since unexplained high GP73 serum concentration was observed in patients with chronic HBV infection, this suggested that soluble GP73 may be playing a role in disease progression. This histological information indicated that non parenchymal cells may be another source of serum GP73. The present interpretation to serum GP73 levels is that HBV replication might increase GP73 secretion, and inflammation might result in GP73 releasing from hepatocytes. The molecular mechanism of GP73 mediating hepatic stellate cells proliferation needed to further elucidated. The main defects of our study is that patients received liver biopsy did not perform liver stiffness measurement, or *vice versa*, since most patients was willing to undertake FinroScan test, rather than liver biopsy. In fact, only thirteen patients received liver biopsy and liver stiffness measurements. We did not perform analysis to those patients separately.

In summary, GP73 may be a useful marker for liver fibrosis grading, especially for diagnosing significant fibrosis and cirrhosis in patients with chronic HBV infections.
